# Supplementary material for: Adenovirus DNA Polymerase Loses Fidelity on a Stretch of Eleven Homocytidines during Pre-GMP Vaccine Preparation
Source: Vaccines (Basel). 2022 Jun 16;10(6):960. doi: 10.3390/vaccines10060960 (PMC9227658; doi:10.3390/vaccines10060960)
Supplement: Supplementary file 1 [file vaccines-10-00960-s001.zip › vaccines-1673713-supplementary.pdf]

DHFR-C11-Pk

ATGATCAGTCTGATTGCGGCGTTAGCGGTAGATCGCGTTATCGGCATGGAAAACGCCATGCCGTGGAACCTGCCTGC
CGATCTCGCCTGGTTTAAACGCAACACCTTAAATAAACCCGTGATTATGGGCCGCCATACCTGGGAATCAATCGGTCG
TCCGTTGCCAGGACGCAAAAATATTATCCTCAGCAGTCAACCGGGTACGGACGATCGCGTAACGTGGGTGAAGTCGG
TGGATGAAGCCATCGCGGCGTGTGGTGACGTACCAGAAATCATGGTGATTGGCGGCGGTCGCGTTTATGAACAGTTC
TTGCCAAAAGCGCAAAAACTGTATCTGACGCATATCGACGCAGAAGTGGAAGGCGACACCCATTTCCCGGATTACGA
GCCGGATGACTGGGAATCGGTATTCAGCGAATTCCACGATGCTGATGCGCAGAACTCTCACAGCTATTGCTTTGAGAT
TCTGGAGCGGCGGGAGCCCAGCG**CCCCCCCCCCC**ATCGTGCAGAACATC**CCCAACCCCCTGCTGGGCCTGGACGTA**C
CCATACGATGTTCCAGATTACGCTGGAACAAAAACTCATCTCAGAAGAGGATCTGTAA

DHFR-C12-Myc

ATGATCAGTCTGATTGCGGCGTTAGCGGTAGATCGCGTTATCGGCATGGAAAACGCCATGCCGTGGAACCTGCCTGC
CGATCTCGCCTGGTTTAAACGCAACACCTTAAATAAACCCGTGATTATGGGCCGCCATACCTGGGAATCAATCGGTCG
TCCGTTGCCAGGACGCAAAAATATTATCCTCAGCAGTCAACCGGGTACGGACGATCGCGTAACGTGGGTGAAGTCGG
TGGATGAAGCCATCGCGGCGTGTGGTGACGTACCAGAAATCATGGTGATTGGCGGCGGTCGCGTTTATGAACAGTTC
TTGCCAAAAGCGCAAAAACTGTATCTGACGCATATCGACGCAGAAGTGGAAGGCGACACCCATTTCCCGGATTACGA
GCCGGATGACTGGGAATCGGTATTCAGCGAATTCCACGATGCTGATGCGCAGAACTCTCACAGCTATTGCTTTGAGAT
TCTGGAGCGGCGGGAGCCCAGCG**CCCCCCCCCCCC**ATCGTGCAGAACATCCCCAACCCCCTGCTGGGCCTGGACGTAC
CCATACGATGTTCCAGATTACGCTG**GAACAAAAACTCATCTCAGAAGAGGATCTG**TAA

DHFR-C13-HA

ATGATCAGTCTGATTGCGGCGTTAGCGGTAGATCGCGTTATCGGCATGGAAAACGCCATGCCGTGGAACCTGCCTGC
CGATCTCGCCTGGTTTAAACGCAACACCTTAAATAAACCCGTGATTATGGGCCGCCATACCTGGGAATCAATCGGTCG
TCCGTTGCCAGGACGCAAAAATATTATCCTCAGCAGTCAACCGGGTACGGACGATCGCGTAACGTGGGTGAAGTCGG
TGGATGAAGCCATCGCGGCGTGTGGTGACGTACCAGAAATCATGGTGATTGGCGGCGGTCGCGTTTATGAACAGTTC
TTGCCAAAAGCGCAAAAACTGTATCTGACGCATATCGACGCAGAAGTGGAAGGCGACACCCATTTCCCGGATTACGA
GCCGGATGACTGGGAATCGGTATTCAGCGAATTCCACGATGCTGATGCGCAGAACTCTCACAGCTATTGCTTTGAGAT
TCTGGAGCGGCGGGAGCCCAGCG**CCCCCCCCCCCCC**ATCGTGCAGAACATCCCCAACCCCCTGCTGGGCCTGGACG**TA**
CCCATACGATGTTCCAGATTACGCTGGAACAAAAACTCATCTCAGAAGAGGATCTGTAA

(a)

INPUT	DHFR-C11-Pk	DHFR-C12-Myc	DHFR-C13-HA
No addition	**11 C's**<br>**198 aa**<br>**22.39 kDa**<br>MISLIAALAVDRVIGMENAM<br>PWNLPADLAWFKRNTLNKP<br>VIMGRHTWESIGRPLPGRKN<br>IILSSQPGTDDRVTWVKSVDE<br>AIAACGDVPEIMVIGGGRVYE<br>QFLPKAQKLYLTHIDAEVEGD<br>THFPDYEPDDWESVFSEFHD<br>ADAQNSHSYCFEILERREPSA<br>**PPP**IVQNI**PNPLLGLD**VPIRCS<br>RLRWNKNSSQKRI	**12 C's**<br>**199 aa**<br>**22.43 kDa**<br>MISLIAALAVDRVIGMENAM<br>PWNLPADLAWFKRNTLNKP<br>VIMGRHTWESIGRPLPGRKN<br>IILSSQPGTDDRVTWVKSVDE<br>AIAACGDVPEIMVIGGGRVYE<br>QFLPKAQKLYLTHIDAEVEGD<br>THFPDYEPDDWESVFSEFHD<br>ADAQNSHSYCFEILERREPSA<br>**PPP**HRAEHPQPPAGPGRTHT<br>MFQITL**EQKLISEEDL**	**13 C's**<br>**199 aa**<br>**22.43 kDa**<br>MISLIAALAVDRVIGMENAM<br>PWNLPADLAWFKRNTLNKP<br>VIMGRHTWESIGRPLPGRKN<br>IILSSQPGTDDRVTWVKSVDE<br>AIAACGDVPEIMVIGGGRVYE<br>QFLPKAQKLYLTHIDAEVEGD<br>THFPDYEPDDWESVFSEFHD<br>ADAQNSHSYCFEILERREPSA<br>**PPP**PSCRTSPTPCWAWT**YPY**<br>**DVPDYA**GTKTHLRRGS
One C added	**12 C's**<br>**207 aa**<br>**23.34 kDA**<br>MISLIAALAVDRVIGMENAM<br>PWNLPADLAWFKRNTLNKP<br>VIMGRHTWESIGRPLPGRKN<br>IILSSQPGTDDRVTWVKSVDE<br>AIAACGDVPEIMVIGGGRVYE<br>QFLPKAQKLYLTHIDAEVEGD<br>THFPDYEPDDWESVFSEFHD<br>ADAQNSHSYCFEILERREPSA<br>**PPP**HRAEHPQPPAGPGRTHT<br>MFQITL**EQKLISEEDL**MRIPGI<br>LE	**13 C's**<br>**208 aa**<br>**23.44 kDA**<br>MISLIAALAVDRVIGMENAM<br>PWNLPADLAWFKRNTLNKP<br>VIMGRHTWESIGRPLPGRKN<br>IILSSQPGTDDRVTWVKSVDE<br>AIAACGDVPEIMVIGGGRVYE<br>QFLPKAQKLYLTHIDAEVEGD<br>THFPDYEPDDWESVFSEFHD<br>ADAQNSHSYCFEILERREPSA<br>**PPP**PSCRTSPTPCWAWT**YPY**<br>**DVPDYA**GTKTHLRRGSVMRI<br>PGILE	**14 C's**<br>**208 aa**<br>**23.51 kDA**<br>MISLIAALAVDRVIGMENAM<br>PWNLPADLAWFKRNTLNKP<br>VIMGRHTWESIGRPLPGRKN<br>IILSSQPGTDDRVTWVKSVDE<br>AIAACGDVPEIMVIGGGRVYE<br>QFLPKAQKLYLTHIDAEVEGD<br>THFPDYEPDDWESVFSEFHD<br>ADAQNSHSYCFEILERREPSA<br>**PPP**PIVQN**IPNPLLGLD**VPIRC<br>SRLRWNKNSSQKRILMRIPGI<br>LE
Two C's added	**13 C's**<br>**223 aa**<br>**25.24 kDa**<br>MISLIAALAVDRVIGMENAM<br>PWNLPADLAWFKRNTLNKP<br>VIMGRHTWESIGRPLPGRKN<br>IILSSQPGTDDRVTWVKSVDE<br>AIAACGDVPEIMVIGGGRVYE<br>QFLPKAQKLYLTHIDAEVEGD<br>THFPDYEPDDWESVFSEFHD<br>ADAQNSHSYCFEILERREPSA<br>**PPP**PSCRTSPTPCWAWT**YPY**<br>**DVPDYA**GTKTHLRRGSNEDP<br>GNSRVRLTCRRPLIKVVRPR	**14 C's**<br>**224 aa**<br>**25.40 kDa**<br>MISLIAALAVDRVIGMENAM<br>PWNLPADLAWFKRNTLNKP<br>VIMGRHTWESIGRPLPGRKN<br>IILSSQPGTDDRVTWVKSVDE<br>AIAACGDVPEIMVIGGGRVYE<br>QFLPKAQKLYLTHIDAEVEGD<br>THFPDYEPDDWESVFSEFHD<br>ADAQNSHSYCFEILERREPSA<br>**PPP**PIVQN**IPNPLLGLD**VPIRC<br>SRLRWNKNSSQKRICNEDPG<br>NSRVRLTCRRPLIKVVRPR	**15 C's**<br>**224 aa**<br>**25.34 kDA**<br>MISLIAALAVDRVIGMENAM<br>PWNLPADLAWFKRNTLNKP<br>VIMGRHTWESIGRPLPGRKN<br>IILSSQPGTDDRVTWVKSVDE<br>AIAACGDVPEIMVIGGGRVYE<br>QFLPKAQKLYLTHIDAEVEGD<br>THFPDYEPDDWESVFSEFHD<br>ADAQNSHSYCFEILERREPSA<br>**PPP**PHRAEHPQPPAGPGRTH<br>TMFQITL**EQKLISEEDL**NEDP<br>GNSRVRLTCRRPLIKVVRPR

(b)

Supplementary Figure S1. Open reading frames and their predicted protein products used in the *in vitro* transcription/translation assay. Homocytidine runs are shown in bold. (**a**) Open reading frames of the input plasmids containing 11-, 12- and 13 -cytidine runs. (**b**) The predicted translated products of the three input plasmids and their derivatives with one or two additional cytidines in the homocytidine run. DFHR - dihydrofolate reductase. Amino acids of the Pk (also known as SV5 – IPNPLLGLD), Myc (EQKLISEEDL) and HA (YDVPDYA) tags are colour coded. The homocytidine stretch is highlighted (yellow box).
